# Long-term remission of type 2 diabetes after very-low-calorie restriction and related predictors

**DOI:** 10.3389/fendo.2022.968239

**Published:** 2022-09-12

**Authors:** Jie Wei, Jie Chen, Xiao Wei, Xiaoduo Xiang, Qing Cheng, Jiechao Xu, Shuhang Xu, Guofang Chen, Chao Liu

**Affiliations:** ^1^ Department of Endocrinology, Affiliated Hospital of Integrated Traditional Chinese and Western Medicine, Nanjing University of Traditional Chinese Medicine, Nanjing, China; ^2^ Department of Nutrition, Affiliated Hospital of Integrated Traditional Chinese and Western Medicine, Nanjing University of Traditional Chinese Medicine, Nanjing, China

**Keywords:** very low-calorie restriction, type 2 diabetes mellitus, long-term remission rate, predictors, diabetes remission

## Abstract

**Purpose:**

Very low-calorie restriction (VLCR) can induce remission of type 2 diabetes mellitus (T2DM), but its long-term remission and related predictors have not been clarified. The aim of present study is to investigate the effect of VLCR in inducing long-term T2DM remission, and the underlying predictors.

**Methods:**

A total of 61 participants with T2DM who received 9 days of VLCR from Dec 2012 to Oct 2020 were followed up in Nov 2021, and divided into responders and non-responders groups. Responders were defined as HbA1c < 6.5% over at least 3 months in the absence of pharmacotherapy. Clinical characteristics were compared between responders and non-responders. Potential predictors were examined by logistic regression analyses based on clinical data before and after VLCR.

**Results:**

Forty-four participants were successfully followed up, including 19 males and 25 females. Long-term remission was observed in 17 participants (38.64%) after VLCR, with a median 7.83 years. Compared with non-responders, responders had a shorter disease duration, a lower fasting blood glucose (FBG) level, a higher fasting insulin level, and better HOMA-β after VLCR. Besides, acute insulin response (AIR), insulin area under curve in intravenous and oral glucose tolerance test (IVGTT-IAUC and OGTT-IAUC) in responders were higher than those in non-responders after VLCR. Multivariable logistic analysis showed that higher post-VLCR IVGTT-IAUC predicted a longer T2DM remission.

**Conclusions:**

After VLCR, more than one third of the participants presented remission over up to 8 years. The improvement of β-cell function, especially the restoration of first-phase insulin-secreting capacity, could prolongate the remission.

## Highlights

This is the first study in which T2DM remission after VLCR is evaluated during the long-term (medium 7y) follow-up. And, there are few studies on predictors of long-term remission of type 2 diabetes after calorie restriction. To our knowledge, the present study described the longest-term T2DM remission after VLCR with a median of 7.83 years follow-up. Our findings provide evidence for considered long-term remission by VLCR as a realistic management target for every newly onset T2DM patients.

## Introduction

Type 2 diabetes mellitus (T2DM) has shown a prevalence increasing rapidly worldwide, which was estimated to be 10.2% in 2021 and reach 11.3% by 2030 in the adult population, and consumed 11.5% of total global health expenditure (1). A recent nationwide analysis in England showed that T2DM was independently associated with a higher odds of in-hospital death with COVID-19 (2). Various strategies have been proposed to induce the remission of T2DM.

In the management of T2DM, focus has shifted to reversing the disease progression, rather than treating symptoms and subsequent consequences (3). The United Kingdom has recommended remission of T2DM as a guideline for T2DM treatment (4). According to the latest Consensus Report by the American Diabetes Association (ADA), the blood glucose level could be normalized after pharmacological or lifestyle intervention, and sustain after withdrawal of glucose-lowering drugs in some cases with diabetes, a condition defined as T2DM remission (5). In 2020, a Position Statement from the American College of Lifestyle Medicine points out that remission should be the goal in the management of T2DM, which can be realized through intensive lifestyle modifications. A weighted average remission rate of 49.4% for T2DM could be achieved after very low-calorie restriction (VLCR, 600-1100 kcal/d) (6). The National Health Service in England has introduced the routine use of VLCR to induce T2DM remission in obese patients (7). The ADA Standards of Medical Care has listed it as a guidance to prescribe VLCR in controlling glycemic level and promote diabetic remission (8).

The long-term remission rate of T2DM after VLCR has been investigated. A cross-sectional study from Scotland in 2019 reported that 4.8% of patients with T2DM achieved remission, manifested as HbA1c < 6.5% in the absence of glucose-lowering therapy for at least 12 months (9). A 2-year remission rate of 35.6% after VLCR was achieved in the Diabetes Remission Clinical Trial (DiRECT), which performed the longest follow-up for analyzing T2DM remission (10). In addition, clinical studies have been carried out for exploring predictors of long-term T2DM remission. A subgroup analysis of DiRECT found that early weight loss (4-8 weeks during intervention) was the strongest predictor of T2DM remission within 1 or 2 years, whereas duration, baseline weight, fasting insulin (FINS), and C-peptide levels were not predictive (11). However, this study was conducted in a population with diabetes duration shorter than 6 years, and predominant white Europeans. No studies have been conducted to explore predictors of post-VLCR T2DM remission in a longer term or in other ethnic groups. Against this background, we conduct this trial to analyze the long-term remission rate after VLCR in Chinese T2DM patients and identify related predictors.

## Methods

### Study design and participants

This is a prospective interventional trial. Inclusion criteria included age of 18- 65 years, body mass index (BMI) of 18.5-40.0 kg/m^2^, diagnosed as T2DM (according to 1999 WHO criteria) with fasting blood glucose (FBG) ≤ 16.7 mmol/L and HbA1b ≤ 10%, and strict compliance with the trial requirements. Exclusion criteria included weight loss > 6 kg in the last 6 months, current use of diet pills, malnutrition, alcoholism, pregnancy or preparation for pregnancy, lactation period, history of gastrointestinal surgery, acute or severe chronic complications of diabetes, acute or chronic pancreatitis, severe chronic infection, hyperuricemia, hyperthyroidism, severe cardiac, renal, hepatic, neurological or psychiatric disease, advanced or extensive metastatic malignancy, or other conditions not suitable for the trial considered by the investigators.

A total of 61 T2DM participants were enrolled and had completed a 9-day VLCR treatment from Dec 2012 to Oct 2020 at the Affiliated Hospital of Integrated Traditional Chinese and Western Medicine, Nanjing University of Traditional Chinese Medicine. At the end of VLCR, nine participants were excluded because their blood glucose failed to reach the target (FBG < 7.0 mmol/L and 2 hours postprandial blood glucose [2hPBG] < 10 mmol/L). In addition, six participants were lost to follow-up. Two participants were eliminated, one due to receiving metabolic surgery after VLCR, and the other due to pregnancy at the time of follow-up. Ultimately, 44 participants were qualified to enter the final analysis. Among them, 29 patients received VLCR from Dec 2012 to Dec 2013, and the remaining 15 patients received this intervention from Jun 2018 to Oct 2020.

### VLCR and follow-up programme

Participants underwent 9 days of VLCR (300-600 kcal/d) according to nutrient ratios set in the 2011 Dietary Guidelines for Chinese Residents. According to the dietary guidelines for Chinese, dietitians formulated an individualized diet for the subjects, with the proportions of nutrients as 55% carbohydrate, 25% fat and 20% protein. During the treatment of VLCR, all participants did not use anti-diabetic and anti-hypertensive drugs, but took in 2-3L of water daily and made only basic activities without intensive physical exercise. Besides, levocarnitine injection (2 g, twice a day) was administered intravenously to promote lipolysis.

At the end of VLCR, participants with normal blood glucose entered the follow-up stage, and those still with high blood glucose received sequential glucose-lowering drugs. According to the guideline for the prevention and treatment of type 2 diabetes mellitus in China, long-term dietary and exercise interventions were recommended for all subjects who completed the 9-day VLCR intervention. All participants were encouraged to modify their lifestyles, such as higher consumption of fish, whole grains and lean meat, and keep doing exercise 150 minutes a week.

Remission of diabetes was defined as hemoglobin A1C (HbA1c) < 6.5% over at least 3 months after cessation of glucose-lowering pharmacotherapy. Participants who achieved diabetes remission were called responders, and non-responders otherwise. All participants were followed up for a median of 7.83 years, and HbA1c was measured to distinguish responders from non-responders.

### Observational parameters and laboratory analyses

Anthropometric and metabolic parameters, including body weight, waist circumference (Wc), blood pressure (BP), glucose and lipid levels, were assessed. Body composition, including protein, skeletal muscle, fat mass, percentage of body fat and visceral fat mass, were measured using a body composition analyzer (Inbody 720). Oral glucose tolerance test (OGTT) and intravenous glucose tolerance test (IVGTT) were performed to assess β-cell function and insulin resistance. All parameters were evaluated before and after VLCR. In addition, urinary ketone was assessed during VLCR to reflect dietary adherence of the participants. At the last follow-up, all subjects completed the lifestyle questionnaire and scales related to diabetes, including diabetes management self-efficiency scale (DMSES), diabetes specific quality of life scale (DSQLS), and summary of diabetes care action (SDSCA).

Several indexes were used for assessment of insulin sensitivity (by HOMA-IR) and the insulin-secreting capacity of β-cells (by HOMA-β). Acute insulin response (AIR) and insulin area under curve (IAUC) in IVGTT were measured after a glucose challenge in the acute phase, and IAUC in OGTT as in the second phase.

Blood glucose was measured by the hexokinase UV endpoint colorimetric method using Diasys reagents and a Roche Modular SWA biochemistry analyzer. Triglyceride was determined by the GPO-POD UV method, total cholesterol by the cholesterol oxidase method, low-density lipoprotein cholesterol and high-density lipoprotein cholesterol by homogeneous enzyme colorimetric method. FINS level was measured by electrochemiluminescence, and HbA1c level by high-pressure liquid chromatography.

### Statistical analysis

Statistical analysis was performed using SPSS 24.0, and continuous variables normally distributed were expressed as mean ± standard deviation, while those not normally distributed as median (upper and lower quartiles). A χ^2^ test was used to compare dichotomous outcomes, and data in normal distribution were analyzed using the independent samples t-test, and the non-parametric test otherwise. Cronbach’s α coefficients of DMSES, DSQLS and SDSCA were calculated to evaluate the internal reliability of the scales. Cronbach’s α coefficient was aquired after the reverse question was processed with “change sign” and then summarized with the forward question. The optimal cut-off value was identified according to the area under curve (AUC) of receiving operating characteristic (ROC). Univariate and multivariate logistic regression analyses were performed to assess odds ratio (OR) and 95% confidence interval (CI). The significant parameters in the univariate analysis were introduced into the multivariate analysis. *P*<0.05 was considered statistically significant.

### Ethics

The study was reviewed and approved by the Ethics Committee of Jiangsu Province Hospital on Integration of Chinese and Western Medicine following national guidelines. All participants provided written informed consent. The trial was registered at Chinese Clinical Trial Registry (No. ChiCTR-TRC-12003512).

## Results

### Baseline characteristics of the participants

**Figure 1:**
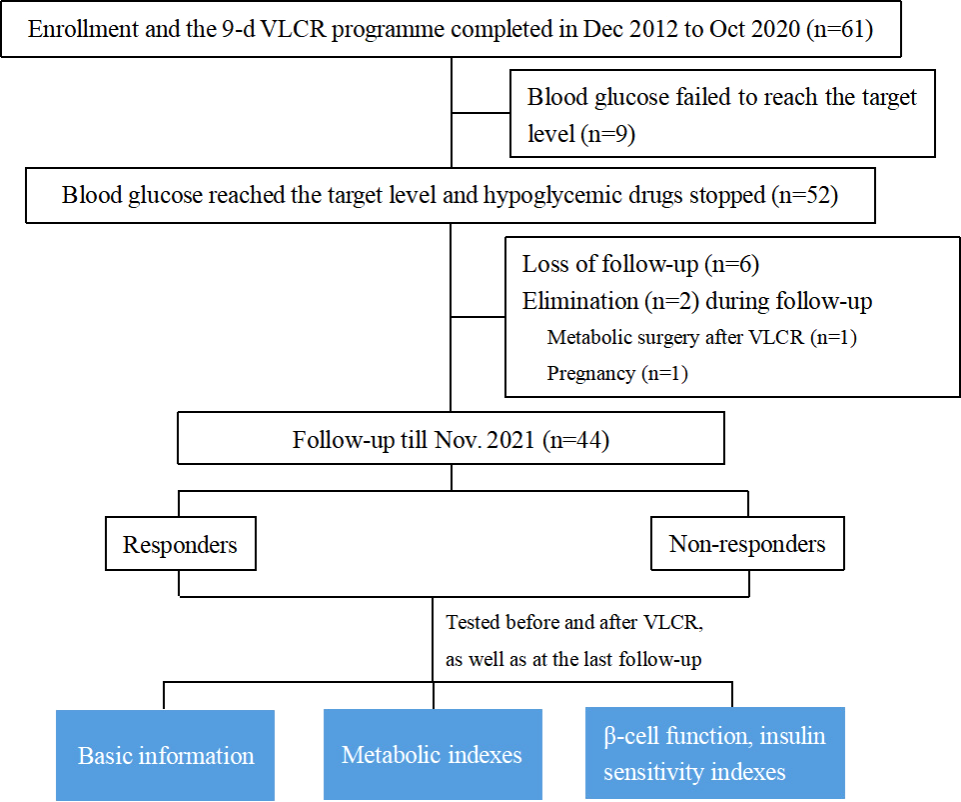
Study flow chart: Enrollment, drop-out and retention. VLCR, very low-calorie restriction; Wc, waist circumstance; WHR, waist-hip-rate; AIR, acute insulin response; IAUC in IVGTT, insulin area under curve in intravenous glucose tolerance test; IAUC in OGTT, insulin area under curve in oral glucose tolerance test.

**Figure 1** shows the study flow chart, including enrollment, drop-out and retention. A total of 44 participants were included, including 19 males, and 25 females. The median age was 45.00 (32.25, 49.00) years, the mean BMI was (27.33 ± 3.37) kg/m^2^, the diabetes duration was 5.00 (0.00, 34.00) months, and the baseline HbA1c was 8.50 (7.33, 9.95) %. Seventeen (38.64%) participants achieved remission over a median of 7.83 (2.81, 8.33) years (responders). Baseline characteristics of all participants were shown in **Table 1**.

**Table 1:** Baseline characteristics of the participants.

Parameters		n	Mean ± SD/Median (upper and lower quartiles)
Age		44	45.00 (32.25, 49.00)
Gender (male) (%)		44	19 (43.18%)
Duration (months)		44	5.00 (0.00, 34.00)
Systolic blood pressure (mmHg)		44	126.00 (120.00, 133.00)
Diastolic blood pressure (mmHg)		44	80.00 (76.00, 89.25)
Body weight (kg)		44	77.62 ± 12.97
BMI (kg/m^2^)		44	27.33 ± 3.37
Waist circumstance (cm)		44	95.41 ± 9.53
HbA1c (%)		44	8.50 (7.33, 9.95)
Fasting blood glucose (mmol/L)		44	8.74 ± 1.71
2-hour postprandial blood glucose (mmol/L)		38	15.88 ± 4.07
Fasting insulin (μU/mL)		37	12.31 ± 5.16
Total cholesterol (mmol/L)		44	4.95 (4.26, 5.67)
Triglyceride (mmol/L)		44	2.06 (1.41, 3.14)
LDL-c (mmol/L)		44	3.15 ± 0.90
HDL-c (mmol/L)		44	1.01 ± 0.25
Body composition	Protein (kg)	15	9.00 (7.80, 9.60)
	Fat mass(kg)	15	21.87 ± 5.21
	Skeletal muscle (kg)	15	25.10 (21.70, 26.80)
	Percentage of body fat (%)	15	31.13 ± 5.51
	Visceral fat mass (cm^2^)	17	97.69 ± 26.13

### Comparison of clinical characters in responders and non-responders

#### Pre and post-VLCR characteristics of responders and non-responders

Before VLCR, responders had a shorter disease duration than non-responders (0.5 [0.00, 12.00] vs. 11.00 [1.00, 36.00] months, *P*=0.049). No significant differences were observed in anthropometric and metabolic parameters at baseline between responders and non-responders, including body weight, BMI, Wc, BP, FBG, 2hPBG, HbA1c, FINS, lipid metabolism parameters (TC, TG, LDL-c, HDL-c) and parameters of β-cell function and insulin sensitivity (HOMA indexes, AIR, IAUC in IVGTT and OGTT) (**Table 2**).

**Table 2:** Pre and post-VLCR anthropometric parameters in responders and non-responders.

Parameters	Responders (n=17)			Non-responders (n=27)		
		Baseline	Post-VLCR	Changes	Baseline	Post-VLCR	Changes
Gender (male/female)	8/9	/	/	11/16	/	/
Age (year)	38.94 ± 9.07	/	/	43.15 ± 9.65	/	/
Duration (months)	0.5 (0.00, 12.00)^*^	/	/	11.00 (1.00, 36.00)	/	/
SBP (mmHg)	133.65 ± 16.92	115.00 ± 9.37	-14.38 ± 16.86	126.48 ± 12.24	116.78 ± 8.82	-9.70 ± 9.15
DBP (mmHg)	86.76 ± 11.48^*^	74.85 ± 10.64	-9.08 ± 11.32	81.00 ± 9.39	72.22 ± 9.13	-8.78 ± 7.66
Body weight (kg)	78.61 ± 13.59	74.31 ± 13.23	-4.29 ± 2.19	77.00 ± 12.78	72.37 ± 12.18	-4.02 ± 1.72
BMI (kg/m^2^)	27.78 ± 3.51	26.23 ± 3.22	-1.54 ± 0.84	27.05 ± 3.32	25.44 ± 3.00	-1.45 ± 0.69
Wc (cm)	97.32 ± 9.27	91.10 ± 8.73	-5.80 ± 3.21	94.20 ± 9.66	89.20 ± 9.39	-4.90 ± 2.33

^*^P<0.05, responders vs. non-responders at baseline.

After VLCR, the levels of FBG, FINS, HOMA-β, AIR, and IAUC in IVGTT and OGTT demonstrated more significant positive changes in responders than in non-responders (**Tables 3**, **4**). But there were no differences in lipid-metabolism-related parameters between the two groups post-VLCR (**Table 3**). The FBG level fell to (5.47 ± 0.98) mmol/L in responders and (6.66 ± 1.53) mmol/L in non-responders (*P*=0.005). The FINS level in responders was significantly higher than that in non-responders (10.84 ± 7.69 vs. 6.29 ± 3.04 μIU/mL, *P*=0.044). Therefore, responders had a better post-VLCR HOMA-β than non-responders (88.18 [42.96, 238.63] vs. 43.69 [27.69, 59.95], *P*=0.005). The function of β-cells to secrete insulin was improved in both first and second phases.

**Table 3:** Metabolic parameters pre- and post-VLCR in responders and non-responders.

Parameters	Responders (n=17)			Non-responders (n=27)		
	Baseline	Post-VLCR	Changes	Baseline	Post-VLCR	Changes
FBG (mmol/L)	8.41 ± 1.79	5.47 ± 0.98^##^	-2.94 ± 1.78	8.95 ± 1.66	6.66 ± 1.53	-2.29 ± 1.35
2hPBG (mmol/L)	14.83 ± 4.21	10.79 ± 2.44	-2.76 ± 3.38	16.36 ± 3.99	12.90 ± 4.14	-2.65 ± 4.28
HbA1c (%)	8.29 ± 1.28	/	/	9.10 ± 2.16	/	/
Fasting insulin (μU/mL)	13.43 ± 5.53	10.84 ± 7.69^#^	-4.37 ± 5.61	11.78 ± 5.00	6.29 ± 3.04	-5.06 ± 4.51
TC (mmol/L)	5.44 ± 1.46	4.99 ± 1.28	-0.45 ± 1.13	4.78 ± 0.97	4.39 ± 1.16	-0.38 ± 0.85
TG (mmol/L)	2.35 (1.58, 3.41)	1.27 (0.95, 1.97)	-0.88 (-1.60, -0.29)	2.04 (1.18, 3.14)	1.28 (0.89, 1.52)	-0.68 (-1.19, -0.12)
LDL-c (mmol/L)	3.43 ± 0.98	3.34 ± 1.01	-0.09 ± 1.04	2.98 ± 0.82	3.02 ± 1.08	0.05 ± 0.76
HDL-c (mmol/L)	0.94 ± 0.22	0.92 ± 0.19	-0.02 ± 0.16	1.06 ± 0.26	0.97 ± 0.21	-0.08 ± 0.18

^#^P<0.05, ^##^P<0.01, responders vs. non-responders post-VLCR.

**Table 4:** Parameters of β-cell function and insulin sensitivity before and after VLCR in responders and non-responders.

Parameters	Responders (n=17)			Non-responders (n=27)		
	Baseline	Post-VLCR	Changes	Baseline	Post-VLCR	Changes
HOMA-IR	3.98 (3.30, 5.94)	1.88 (1.43, 3.41)	-2.14 (-3.30, -1.49)	4.63 (3.21, 6.53)	1.58 (1.04, 2.70)	-3.04 (-4.11, -0.79)
HOMA-β	53.41 (42.01, 95.70)	88.18 (42.96, 238.63)^##^	13.58 (-27.14, 43.80)	46.51 (27.45, 58.10)	43.69 (27.69, 59.95)	-1.43 (-9.27, 12.85)
AIR (μIU/mL.min)	7.59 ± 5.17	14.38 ± 9.15^#^	6.79 ± 5.89^△△^	5.70 ± 2.31	6.03 ± 2.90	0.45 ± 3.38
IVGTT-IAUC (mU/L)	172.49 ± 113.20	300.85 ± 172.07^#^	128.36 ± 105.29^△△^	130.78 ± 49.11	126.59 ± 55.32	-5.38 ± 66.61
OGTT-IAUC (mU/L)	8263.12 ± 8570.63	10328.82 ± 6051.94^#^	367.85 ± 5794.90	5103.41 ± 3275.41	4619.03 ± 2424.71	1.22 ± 2676.54

^#^P<0.05, ^##^P<0.01, responders vs. non-responders post-VLCR; ^△△^P<0.01, responders vs. non-responders, post-VLCR-to-baseline difference.

**Figure 2:**
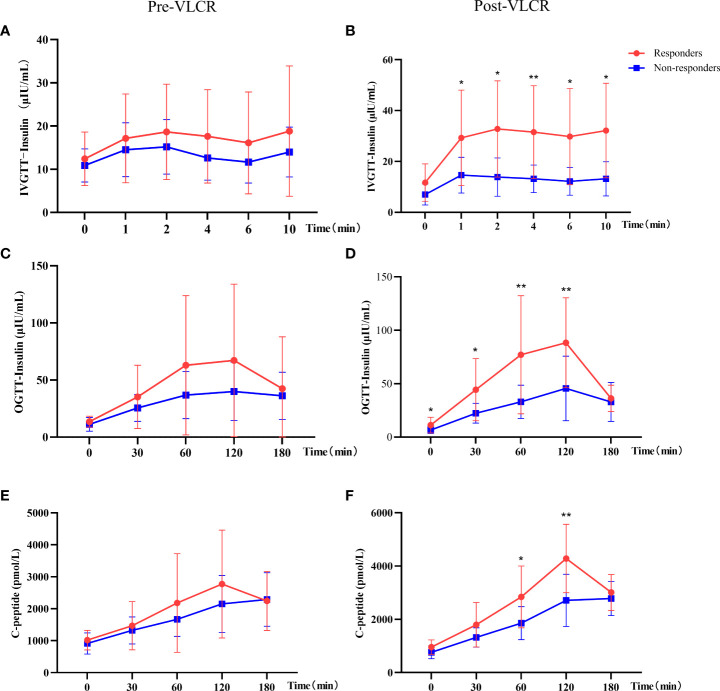
Comparison of insulin and C-peptide in IVGTT and OGTT before and after VLCR in responders and non-responders. **(A, B)**, insulin secretion in IVGTT pre- (left) and post-VLCR (right); **(C, D)**, insulin secretion in OGTT pre- (left) and post-VLCR (right); **(E, F)**, C-peptide secretion in OGTT pre- (left) and post-VLCR (right). ^*^ *P*<0.05, ^**^ *P*<0.01, responders vs. non-responders at different time points.

Specifically, the insulin level was significantly higher in responders than in non-responders at 1 min, 2 min, 4 min, 6 min, and 10 min in IVGTT (**Figures 2A, B**). IAUC in IVGTT increased from 172.49 ± 113.20 μU/L to 300.85 ± 172.07 μU/L in responders, while decreased from 130.78 ± 49.11 μU/L to 126.59 ± 55.32 μU/L in non-responders, therefor IAUC in IVGTT was markedly improved following VLCR in responders than non-responders (128.36 ± 105.29 vs. -5.38 ± 66.61 mU/L, *P*=0.001). Meanwhile, AIR increased in responders from 7.59 ± 5.17 μIU/mL.min to 14.38 ± 9.15 μIU/mL.min (*P*=0.009) after VLCR, whereas no change was observed in the non-responders (5.70 ± 2.31 vs. 6.03 ± 2.90 μIU/mL.min, *P*=0.629). The increasement of AIR in remission group was significantly higher than that in non-remission group (6.79 ± 5.89 vs. 0.45 ± 3.38 μIU/mL.min, *P*=0.003).

In the second phase of insulin secretion by β-cells, responders had a higher insulin level at 0 min, 30 min, 60 min and 120 min than non-responders in OGTT after VLCR (**Figures 2C, D**). IAUC in OGTT increased in responders, while decreased in non-responders, and there was a significant difference between two groups (10328.82 ± 6051.94 vs. 4619.03 ± 2424.71 mU/L, *P*=0.011). In addition, there was no difference in C-peptide at every time-point in OGTT between two groups before VLCR, but after VLCR, C-peptide levels at 60 and 120 min were higher in responders than in non-responders (**Figures 2E, F**) (**Table 4**).

There was no statistical difference in protein, skeletal muscle and body fat mass between responders and non-responders after VLCR. However, there were greater decreases in body fat mass and percentage of body fat in responders (-2.40 ± 0.65 vs. -1.56 ± 0.62 kg, *P*=0.030; -1.94 ± 0.65 vs. 0.50 ± 4.93%, *P*=0.040, respectively) (**Table 5**).

**Table 5:** Body composition before and after VLCR in responders and non-responders.

Parameters	Responders (n=5)			Non-responders (n=10)		
	Baseline	Post-VLCR	Changes	Baseline	Post-VLCR	Changes
Protein (kg)	9.10 (7.85, 10.60)	8.90 (7.55, 10.35)	-0.20 (-0.35, -0.20)	9.00 (7.80, 9.68)	8.60 (7.70, 9.38)	-1.00 (-1.35, -0.58)
Skeletal muscle (kg)	25.40 (21.55, 29.90)	24.80 (20.85, 29.30)	-0.50 (-0.95, -0.40)	25.05 (21.68, 27.05)	24.05 (21.25, 26.20)	-1.00 (-1.35, -0.58)
Fat (kg)	21.82 ± 5.47	19.42 ± 4.91	-2.40 ± 0.65^△^	21.89 ± 5.37	20.33 ± 5.00	-1.56 ± 0.62
Body fat percentage (%)	31.90 ± 3.67	29.96 ± 3.96	-1.94 ± 0.65^△^	30.74 ± 6.38	31.24 ± 6.42	0.50 ± 4.93
Visceral fat mass (cm^2^)	105.94 ± 27.80	99.67 ± 20.25	-9.12 ± 11.33	91.92 ± 24.67	85.18 ± 27.25	-6.74 ± 9.74

^△^P<0.05, responders vs. non-responders, post-VLCR-to-baseline difference.

#### Clinical characters at the last follow-up in responders and non-responders

**Figure 3:**
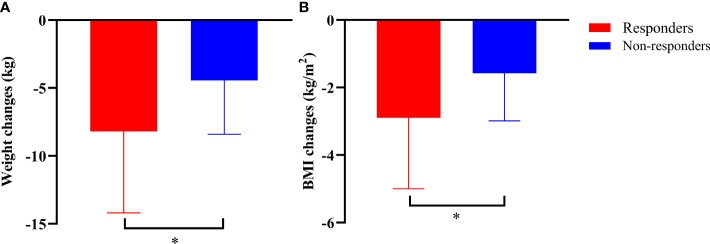
Body weight **(A)** and BMI **(B)** at the last follow-up in responders and non-responders compared to those at baseline. ^*^ *P*<0.05, responders vs. non-responders.

**Figure 4:**
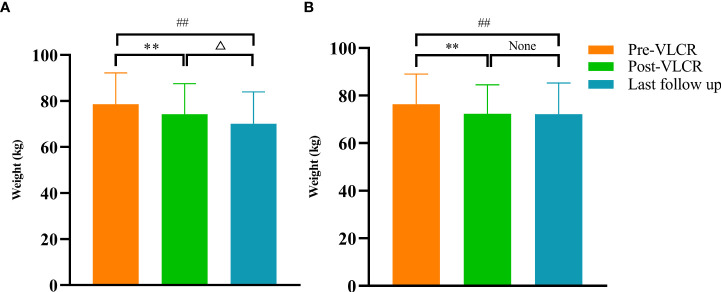
Body weight before and after VLCR, as well as at the last follow-up in responders **(A)** and non-responders **(B)**. ^**^ *P*<0.01, pre vs. post-VLCR; ^##^ *P*<0.01, pre-VLCR vs. last follow-up; ^△^ *P*<0.05, post-VLCR vs. last follow-up.

Compared with those at baseline, the body weight and BMI were significantly reduced in both responders and non-responders at the last follow-up. Responders had a greater reduction in body weight than non-responders (-8.19 ± 6.00 vs. -4.44 ± 3.97 kg, *P*=0.038) (**Figure 3A**). Similarly, the BMI decreased from 27.78 ± 3.51 kg/m^2^ to 25.02 ± 2.95 kg/m^2^ in responders, and from 27.05 ± 3.32 kg/m^2^ to 25.26 ± 3.47 kg/m^2^ in non-responders. Compared with that at baseline, there was a significant difference in BMI between responders and non-responders at the last follow-up (-2.90 ± 2.10 vs. -1.58 ± 1.41 kg/m^2^, *P*=0.022) (**Figure 3B**). Notably, compared with that after VLCR, the body weight in responders decreased further from 74.31 ± 13.23 kg to 71.02 ± 12.07 kg at the last follow-up (*P*=0.020), but this decrease was not observed in non-responders (71.90 ± 12.69 vs. 71.48 ± 12.96 kg, *P*=0.618) (**Figures 4A, B**).

**Figure 5:**
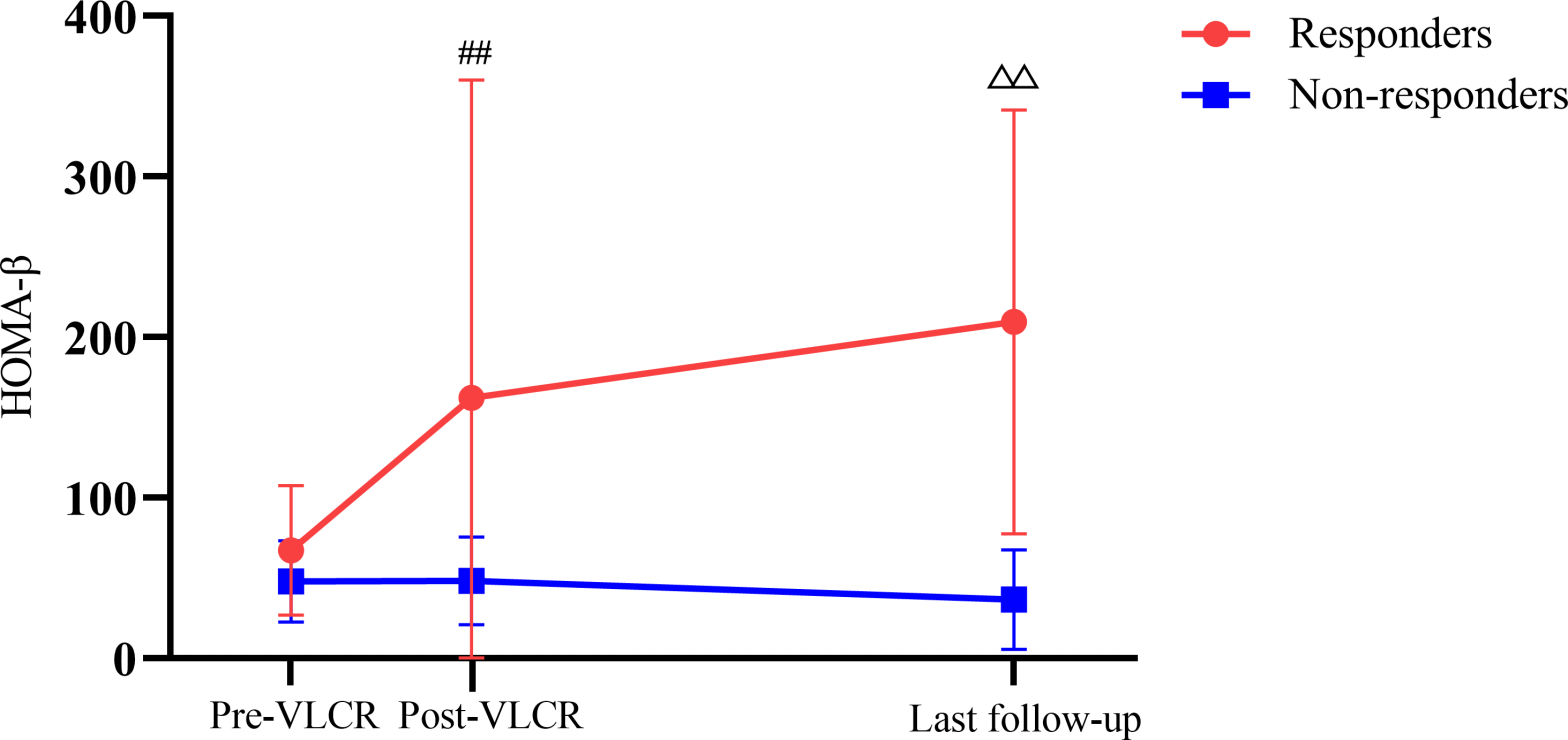
HOMA-β before and after VLCR, as well as at the last follow-up in responders and non-responders. ^##^ *P*<0.01, responders vs. non-responders post-VLCR; ^△△^ *P*<0.01, responders vs. non-responders at the last follow-up.

At the last follow-up, the level of FINS in responders was higher than that in non-responders (15.57 ± 3.81 vs. 8.54 ± 4.71 μU/mL, *P*=0.007), as was HOMA-β (129.11 [116.94, 342.09] vs. 27.58 [15.19, 48.09], *P*=0.001) (**Figure 5**). Anthropometric, metabolic parameters, body composition, and HOMA-IR showed no significant difference between the two groups at the last follow-up (**Tables 6**, **7**).

**Table 6:** Anthropometric parameters at baseline and the last follow-up in responders and non-responders.

Parameters	Responders (n=17)			Non-responders (n=27)		
	Baseline	Last follow-up	Changes	Baseline	Last follow-up	Changes
SBP (mmHg)	133.65 ± 16.92	129.83 ± 16.33	8.00 ± 24.25	126.48 ± 12.24	123.50 ± 12.13	-0.50 ± 5.92
DBP (mmHg)	86.76 ± 11.48^*^	79.67 ± 9.22	-5.83 ± 11.86	81.00 ± 9.39	77.07 ± 8.31	-2.29 ± 6.47
Body weight (kg)	78.61 ± 13.59	71.02 ± 12.07	-8.19 ± 6.00^△^	77.00 ± 12.78	72.19 ± 13.14	-4.44 ± 3.97
BMI (kg/m^2^)	27.78 ± 3.51	25.02 ± 2.95	-2.90 ± 2.10^△^	27.05 ± 3.32	25.26 ± 3.47	-1.58 ± 1.41
Wc (cm)	97.32 ± 9.27	89.20 ± 7.82	-5.30 ± 6.04	94.20 ± 9.66	89.42 ± 7.74	-0.79 ± 5.44

^*^P<0.05, responders vs. non-responders at baseline. ^△^P<0.05, responders vs. non-responders, difference between follow-up and baseline parameters.

**Table 7:** Metabolic parameters at baseline and the last follow-up in responders and non-responders.

Parameters	Responders (n=17)			Non-responders (n=27)		
	Baseline	Last follow-up	Changes	Baseline	Last follow-up	Changes
FBG (mmol/L)	8.41 ± 1.79	5.31 ± 0.78^##^	-2.56 ± 2.95^△^	8.95 ± 1.66	10.10 ± 3.94	1.08 ± 3.89
2hPBG (mmol/L)	14.83 ± 4.21	8.87 ± 3.97	-1.01 ± 1.33	16.36 ± 3.99	16.63 ± 3.62	-1.05 ± 4.66
HbA1c (%)	8.29 ± 1.28	5.63 ± 0.53	-2.97 ± 2.26	9.10 ± 2.16	8.41 ± 1.98	-0.56 ± 2.42
FINS (μU/mL)	13.43 ± 5.53	15.57 ± 3.81^##^	2.96 ± 4.70	11.78 ± 5.00	8.54 ± 4.71	-1.54 ± 4.52
TC (mmol/L)	5.44 ± 1.46	4.79 ± 0.97	-1.04 ± 1.88	4.78 ± 0.97	4.67 ± 1.22	0.01 ± 1.13
TG (mmol/L)	2.35 (1.58, 3.41)	1.27 (0.95, 1.97)	-0.93 (-5.70, 0.06)	2.04 (1.18, 3.14)	1.28 (0.89, 1.52)	-0.49 (-1.54, 0.47)
LDL-c (mmol/L)	3.43 ± 0.98	3.06 ± 1.06	-0.08 ± 1.03	2.98 ± 0.82	3.20 ± 1.17	0.32 ± 0.88
HDL-c (mmol/L)	0.94 ± 0.22	1.21 ± 0.26	0.27 ± 0.13	1.06 ± 0.26	1.17 ± 0.24	0.13 ± 0.24
HOMA-IR	3.98 (3.30, 5.94)	4.14 (2.52, 4.74)	0.37 (-0.95, 1.56)	4.63 (3.21, 6.53)	3.48 (2.51, 4.36)	-0.22 (-1.77, 0.47)

^##^P<0.01, responders vs. non-responders at the last follow-up; ^△^P<0.05, responders vs. non-responders, difference between follow-up and baseline parameters.

In the lifestyle questionnaire, at last follow-up, compared with non-responders, the responders consumed significantly lower raw weight of grain foods such as rice, noodles and multigrains per week, and more fresh vegetable consumption per week (1.16 ± 0.72 vs. 2.04 ± 0.76 kg, *P*=0.036; [2.45 (2.10, 4.20) vs. 1.75 (0.70, 2.10), *P*=0.034], respectively). And, there were no significant differences in the diabetes-related scales between responders and non-responders (DMSES: [170.00 (167.50, 191.25) vs. 160.00 (142.00, 168.00), *P*=0.186; DSQLS: [45.00 (40.00, 50.00) vs. 44.00 (41.50, 53.50), *P*=0.413]; SDSCA: 47.25 ± 4.50 vs. 38.08 ± 14.39, *P*=0.064). Though, for diet and exercise management of SDSCA, the total scores were both significantly higher in responders than non-responders (11.75 ± 2.22 vs. 6.92 ± 4.19, *P*=0.046; 10.25 ± 1.26 vs. 6.77 ± 5.17, *P*=0.042, respectively) (**Table 8**).

**Table 8:** Lifestyle questionnaire and diabetes-related scales at last follow-up in responders and non-responders.

Items	Responders	Nonresponders	*P* value
Raw weight of food grains (kg/week)	1.16 ± 0.72	2.04 ± 0.76	0.036
Fresh vegetables (kg/week)	2.45 (2.10, 4.20)	1.75 (0.70, 2.10)	0.034
Number of eggs (kg/week)	7.00 ± 0.00	10.23 ± 4.57	0.197
DMSES	170.00 (167.50, 191.25)	160.00 (142.00, 168.00)	0.186
DSQLS	45.00 (40.00, 50.00)	44.00 (41.50, 53.50)	0.413
SDSCA	47.25 ± 4.50	38.08 ± 14.39	0.064
Diet management	11.75 ± 2.22	6.92 ± 4.19	0.046
Exercise management	10.25 ± 1.26	6.77 ± 5.17	0.042

### Predictors for long-term T2DM remission

ROC curves were depicted to identify the optimal cut-off values of continuous variables. As shown by univariate logistic analysis (**Table 9**), the following post-VLCR parameters were related to T2DM remission, including FINS ≥ 5.075 μIU/mL (OR: 1.23, 95% CI: 1.04-1.55, *P*=0.042), HOMA-β ≥ 65.67 (OR: 1.02, 95% CI: 1.01-1.05, *P*=0.034), IAUC in IVGTT ≥ 171.21 mU/L (OR: 1.03, 95% CI: 1.01-1.06, *P*=0.018) and IAUC in OGTT ≥ 4430.63 mU/L (OR: 1.001, 95% CI: 1.000-1.002, *P*=0.011). In addition, the increment of AIR and IAUC in post-VLCR IVGTT were also related to long-term remission of T2DM (ΔAIR ≥ 0.53 μIU/mL.min, OR: 1.41, 95% CI: 1.10-2.04, *P*=0.026; ΔIAUC in IVGTT ≥ 13.97 mU/L, OR: 1.02, 95% CI: 1.01-1.04, *P*=0.016, respectively). Furthermore, FBG ≥ 5.69 mmol/L post-VLCR was an adverse factor for the remission of T2DM (OR: 0.413, 95% CI: 0.18-0.77, *P*=0.017).

**Table 9:** Univariate logistic regression analysis for predictors of diabetes remission.

Parameters	OR	95% CI	*P* value
Duration	0.975	0.938-1.001	0.114
FBG post-VLCR	0.413	0.176-0.769	0.017
FINS post-VLCR	1.226	1.044-1.547	0.042
HOMA-β post-VLCR	1.024	1.007-1.051	0.034
AIR post-VLCR	1.115	0.897-1.431	0.333
IAUC in IVGTT post-VLCR	1.028	1.01-1.059	0.018
IAUC in OGTT post-VLCR	1.001	1-1.002	0.011
△AIR post-VLCR	1.407	1.104-2.043	0.026
△IVGTT in IAUC post-VLCR	1.021	1.007-1.043	0.016
△Body fat post-VLCR	0.100	0.003-0.75	0.080
△Body fat percentage post-VLCR	0.308	0.057-0.977	0.101

The symbol △ means “difference between post-VLCR and pre-VLCR”.

To further identify independent predictors for long-term T2DM remission, we performed multivariable analysis. After adjusted the following parameters, including post-VLCR FBG, FINS and HOMA-β, it was found that higher IAUC in post-VLCR IVGTT was more predictive of T2DM remission (OR: 1.020, 95% CI: 1.002-1.040, *P*=0.032). After further adjustment of post-VLCR BMI and △BMI, IAUC-IVGTT could still predict the long-term remission of T2DM (OR: 1.034, 95% CI: 1.003-1.066, *P*=0.037) (**Table 10**).

**Table 10:** Multivariable logistic regression analysis for predictors of diabetes remission.

Predictors	OR	95% CI		*P* value
Model 1				
FBG post-VLCR	1.143	0.030	43.714	0.943
FINS post-VLCR	0.740	0.113	4.846	0.753
HOMA-β post-VLCR	1.044	0.822	1.324	0.725
IAUC-IVGTT post-VLCR	1.020	1.002	1.040	0.032
Model 2				
BMI post-VLCR	0.998	0.581	1.715	0.994
△BMI post-VLCR	3.571	0.123	103.474	0.459
FBG post-VLCR	0.364	0.0001	2041.694	0.819
FINS post-VLCR	1.259	0.026	61.017	0.907
HOMA-β post-VLCR	0.972	0.593	1.592	0.910
IAUC-IVGTT post-VLCR	1.034	1.002	1.066	0.037

The symbol △ means “difference between post-VLCR and pre-VLCR”.

### Safety during VLCR

Hypoglycemia (capillary blood glucose < 3.9 mmol/L) occurred in 9 patients during VLCR, with a total of 21 occurrences and the maximum number of 3 occurrences in 1 patient. The lowest blood glucose level was 3.1 mmol/L. Hypoglycemia occurred in 6 patients at the 6th day of VCLR, which was the peak time of hypoglycemia. Most patients (35/44) presented with transient elevation of serum uric acid, which decreased during the follow-up after discharge. There were no cases of gout during the period of VLCR. A mild to moderate increase of aminotransferase was observed in 3 patients, with a maximum of 162 U/L for alanine aminotransferase and 72 U/L for aspartate aminotransferase; however, they decreased to 74 U/L and 50 U/L at the end of VLCR, respectively. None had serious adverse events, including severe hypoglycemia, arrhythmia, electrolyte disturbance, ketoacidosis, hemocytopenia or anemia.

## Discussion

The present study described the longest-term T2DM remission after VLCR. Seventeen participants achieved remission over a median of 7.83 years after VLCR, with a remission rate of 38.64%, which suggested that VLCR can achieve long-term T2DM remission. Responders had similar anthropometric and metabolic parameters to non-responders at baseline, except for a shorter diabetes duration. After VLCR, responders presented with lower FBG, a higher FINS level and more positive β-cell function indices, including HOMA-β, AIR, IAUC in IVGTT and OGTT. After VLCR, the increments of AIR and IAUC in IVGTT, and the decrease in body fat mass and body fat percentage were more pronounced in responders. Compared with those at baseline, the decreases in body weight and BMI at the last follow-up were significantly greater in responders than in non-responders. In the univariate logistic regression analysis, post-VLCR FBG, FINS, HOMA-β, IAUC in IVGTT and OGTT were all related to the long-term remission of T2DM. After adjustment for post-VLCR BMI and △BMI, IAUC in IVGTT still showed an obvious positive association with long-term T2DM remission.

Studies lack about the association between T2DM duration with T2DM remission after VLCR. Steven et al. found after 8 weeks of the VLCR, the patients who remained in remission for 6 months had a significantly shorter diabetes duration than those not in remission (12, 13). In addition, the remission rate after 8 weeks of VLCR was 87% in patients with a diabetes duration less than 4 years, but just 50% in patients with a duration more than 8 years (12). Another prospective study explored T2DM remission in 19 overweight adults receiving VLCR of 600 kcal/day for 10 weeks, followed by a 4-week food reintroduction. After 12 weeks of re-feeding, 79% of subjects were still in T2DM remission, and the mean duration was shorter in responders significantly (2 years in responders vs. 6 years in non-responders) (14). The DiRECT sub-group study also found that at 1 year after intervention, responders had a shorter duration than non-responders (2.7 ± 0.3 vs. 3.8 ± 0.4 years) (15). In addition, the Diabetes Intervention Accentuating Diet and Enhancing Metabolism study, which enrolled participants with duration less than 3 years, had a rate of remission at 12 months (60%), higher than that found in DiRECT study (46%) which recruited participants with longer duration (≤ 6 years) (10, 16). These results are consistent with our findings in the present study, suggesting that duration may play an important role in T2DM remission induced by VLCR.

However, our further logistic regression analysis showed that the duration of diabetes was not a predictor of long-term remission T2DM after VLCR, which supports the results of another DiRECT sub-study that the duration could not predict T2DM remission at 1 and 2 years. This might be related to the fact that the DiRECT only included diabetic patients with a disease duration fewer than 6 years (11). In our study, however, 93.18% of the participants presented with a duration less than 6 years. In contrast, the univariate and multivariate logistic regression analyses conducted in the Look AHEAD sub-study found that the duration less than 2 years could predict the remission of T2DM at 2-4 years. However, the intervention protocol of the Look AHEAD study should be noted (17). Its food restriction had a low intensity (1200-1800 kcal/day), but lasted for 4 years and combined with exercise (175 minutes per week). This interventional mode is significantly different from those in our study and DiRECT. In the future, more rigorous VLCR intervention should be performed in patients with different durations of T2DM to further clarify the role of duration in the long-term remission. Undoubtedly, we speculate that the remission duration decreases as T2DM duration increases, and the optimal time to reverse T2DM is at diagnosis.

Partial studies have shown that weight loss could induce T2DM remission in a dose-dependent manner (9, 10, 18, 19). However, in our study, the body weight changes during VLCR did not differ significantly between the two groups, which is consistent with the results from DiRECT sub-study (15). Weight changes before and after VLCR did not predict long-term remission of T2DM, which is not in line with the findings of Thom et al (11). The reason may be that the course of VLCR in our study was short, so the patients could not lose too much weight. Notably, in our study, the magnitude of weight regain (compared with post-VLCR weight) at the last follow-up was significantly greater in non-responders than in responders, suggesting that weight loss and maintenance after VLCR were closely associated with long-term remission. Al-Mrabeh et al. have reported the features of patients who achieved remission through 5 months of weight loss intervention, but T2DM relapsed 2 years later (20). Patients in whom T2DM relapsed regained more weight between 5 months and 2 years, compared to those in long-term remission. In addition, Thom et al. also found that early weight loss (in 4 weeks) was an important predictor for the remission of diabetes, and proposed “stopping rules” for participants who failed to lose weight early (11). Our results complemented the “stopping rules” and stated that weight loss should be maintained in a long term. Therefore, early weight loss and maintenance are both crucial for long-term T2DM remission.

The questionnaire revealed that the consumption of grains in responders was significantly lower, while the consumption of vegetables was higher than non-responders. Although our study did not further to explore the specific classification of grains (such as different glucose index grain) and their share of total daily calories, but for patients with type 2 diabetes, low grain and high vegetable intake is generally recommended healthy eating patterns. It can be concluded that long-term remission of type 2 diabetes is more likely to be achieved after short-term VLCR followed by long-term healthy diet. In addition, diet and exercise scores were higher in responders, meaning that they had more rigorous management than non-responders. Our finding supports Magkos et al’s (21) claim that diet combined with exercise could prevent weight gain and recurrence of type 2 diabetes after remission.

The UK Prospective Diabetes Study has shown that β-cell function begins to decline before T2DM onset and β-cell function recovery is of great significance for T2DM remission (22). A retrospective observational study has found that β-cell function after pharmacotherapy is a strong predictor of T2DM status (23). In 2004, Weir et al. have put forward 5 stages of β-cell dysfunction in the development of diabetes, suggesting that β-cell function could be reversed during the first stage (compensation) to the fourth stage (stable decompensation) (24). In 2011, Lim et al. have demonstrated that VLCR can reverse the acute insulin secretion by β-cells to normal in T2DM (25). Subsequent studies have further found that first-phase insulin secretion increases after weight loss in T2DM patients responding to VLCR, but not in those who do not respond to VLCR, despite of similar weight loss (13, 15). These findings are in accordance with ours, indicating that the long-term T2DM remission depends on the recovery of insulin-secreting capacity of β-cells in the acute phase.

To date, no studies have established whether the recovery of β-cell function after VLCR could predict prolonged remission of T2DM. A recent study has found that AIR recovery following continuous subcutaneous insulin injections could predict the long-term remission of T2DM (26). Besides AIR, in our study, we found that IAUC in IVGTT could serve as a predictive factor for long-term T2DM remission. The above findings demonstrate that the improvement of β-cell function is associated with long-term T2DM remission. Taylor et al. have noted that substantial weight loss and prevention of weight regain help to recover and maintain β-cell function (27). Therefore, evidence-based approaches should be considered for long-term avoidance of weight regain.

Several studies have confirmed that VLCR can reverse T2DM; however, these studies only focus on short to medium-term diabetes remission after VLCR (10, 13, 18, 25). Hence, our study is innovative by demonstrating that plasma glucose can be normalized in a long term by short-term VLCR. Nevertheless, the definition of T2DM remission remains debated. More than 96 definitions of diabetes remission have been used in the literature published from 2009 to 2020 (28). In the present study, the latest definition of T2DM remission was used, which provides a more valuable reference for subsequent related studies (5). Our findings may provide a basis for developing efficient tools to better predict T2DM remission in response to VLCR.

Limitations also exist in our study. First, the sample size was small and our study was conducted at one site, so larger-sample multi-center studies are needed to confirm our findings. Second, our study was based on rigorous dietary intervention, which ensured the quality of the trial, but may not reflect the intensity of compliance in a free-living population during the follow-up. Third, our study was conducted in a population with dominant Chinese and a relatively short disease duration. More evidences are needed to establish the likelihood of remission for people with longer disease durations and other ethnicities. Finally, HOMA-IR cannot accurately reflect insulin resistance in the liver. Thus, future studies should evaluate the influence of calorie restriction on hepatic insulin resistance and complete twin vicious cycles of T2DM (29).

Despite these limitations, this is the first study in which T2DM remission after VLCR is evaluated during the long-term (medium 7y) follow-up. A recent prospective cohort study has demonstrated the potential of primary care in achieving T2DM remission. Having completed the diabetes-specific consultation without dietary changes within the first year after diagnosis, 30% of T2DM patients can achieve remission at 5 years of follow-up (30). Primary care and clinical management are beneficial for the long-term remission of T2DM. Demographic and clinical predictors can be used to select T2DM populations with potential of achieving long-term remission after VLCR. In summary, VLCR can achieve long-term remission in patients with T2DM. The metabolic abnormalities of T2DM can be reversed by short-term VLCR which may be easily translated into clinical practice.

## Data availability statement

The original contributions presented in the study are included in the article/supplementary material. Further inquiries can be directed to the corresponding authors.

## Ethics statement

The studies involving human participants were reviewed and approved by Ethics Committee of Jiangsu Province Hospital on Integration of Chinese and Western Medicine. The patients/participants provided their written informed consent to participate in this study.

## Author contributions

All authors contributed to the study conception and design. Material preparation, data collection and analysis were performed by JW, JC, XX, QC, and JX. Patients enrolled and follow up were performed by XW and SX. The first draft of the manuscript was written by JW and GC, and all authors commented on previous versions of the manuscript. All authors read and approved the final manuscript.

## Acknowledgments

The formula diet of this study was offered by Department of Nutrition, Affiliated Hospital of Integrated Traditional Chinese and Western Medicine, Nanjing University of Traditional Chinese Medicine. We thank Doudou Li, Yalin Wang and Tingting Wang, the Department of Endocrinology, Affiliated Hospital of Integrated Traditional Chinese and Western Medicine for providing questionnaire and diabetes-related scales support. We thank Xingjia Li, Yijiao Xu and Yu Chen from the Department of Endocrinology, Affiliated Hospital of Integrated Traditional Chinese and Western Medicine for their participation and help.

## Conflict of interest

The authors declare that the research was conducted in the absence of any commercial or financial relationships that could be construed as a potential conflict of interest.

## Publisher’s note

All claims expressed in this article are solely those of the authors and do not necessarily represent those of their affiliated organizations, or those of the publisher, the editors and the reviewers. Any product that may be evaluated in this article, or claim that may be made by its manufacturer, is not guaranteed or endorsed by the publisher.
